# Patient experiences and anxiety related to medical imaging: challenges and potential solutions

**DOI:** 10.1002/jmrs.748

**Published:** 2023-12-28

**Authors:** Kristy Fakes

**Affiliations:** ^1^ School of Medicine and Public Health, College of Health, Medicine and Wellbeing University of Newcastle Callaghan New South Wales Australia; ^2^ Hunter Medical Research Institute New Lambton Heights New South Wales Australia

## Abstract

Patients' experiences of medical imaging are varied. In referencing the work of Plunkett et al. https://doi.org/10.1002/jmrs.725, relating to fetal MRI, this editorial explores potential methods for increased education and support to alleviate anxiety in patients undergoing medical imaging procedures.

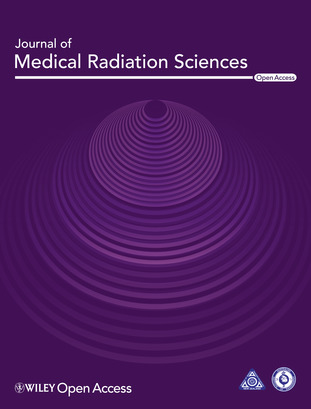

## Patients Experience Medical Imaging in Varied Ways

Patients undergo a range of diagnostic medical interventions during pregnancy, with fetal magnetic resonance imaging (MRI) now a commonly used imaging modality,^1^ complementing ultrasound findings with increased diagnostic accuracy and considered as standard for some high‐risk cases, according to international guidelines.^2^ However, like other imaging procedures,^3^ fetal MRI scans can induce feelings of fear and anxiety.^4^, ^5^

The original article published in this issue by Plunkett et al.,^5^ describes a three‐arm pilot study that explored the impact of information leaflets on anxiety amongst pregnant women referred for fetal MRI. Group A received usual care, Group B received a basic information leaflet, and Group C received a comprehensive information leaflet. Patients reported that both types of leaflets reduced their anxiety.^5^ This editorial further considers recommendations for education and support for the reduction of anxiety in patients undergoing medical imaging procedures including fetal MRI.

## Patient Education and Preparedness

Patient preparation for medical interventions, including those related to diagnosis, is crucial and involves education, support and involvement in decision‐making.^6^ To ensure patients are adequately prepared for what will happen before, during and after a medical intervention, including medical imaging, five content areas are generally recommended^7^: (1) risk communication; (2) procedural information; (3) sensory information; (4) behavioural instruction; (5) psychosocial aspects. For fetal MRI scans this could include *Disease‐specific information* (e.g. what congenital disease is); *Risk communication* (potential benefits, side‐effects and complications of fetal MRI); *Procedural information* (the sequence of events that will occur during the scan); *Sensory information* (what the patient may see, feel or hear during the scan); *Behavioural instruction* (the patients' role in the scan, e.g. lack of movement is required); and *Psychosocial aspects* (strategies to recognise and manage anxiety). Plunkett et al.'s^5^ information leaflet addressed these aspects in some detail. It is suggested that psychosocial aspects be an area of further focus for future interventions.

## Health Literacy

Patients' health literacy remains low,^8^, ^9^ and efficient interventions to enhance patient understanding and outcomes associated with health literacy are needed.^10^ The authors chose an information leaflet as the format for their patient education intervention to improve understanding and help reduce maternal anxiety surrounding fetal MRI. They reported a statistically significant difference (*χ* ^2^(2) = 11.3, *P* = 0.024) between groups in regards to participant agreement that they felt anxious before the fetal MRI, with 50%, 57% and 77% of participants in Group A, B and Group C.^5^ Surprisingly, Group A (usual care), reported the least anxiety, compared to Groups B and C, leading the authors to suggest that providing an information leaflet before a scan may increase confusion and anxiety.^5^ However, the results showed no statistically significant difference between groups in relation to whether the information leaflet made them feel less anxious. Although no statistically significant difference was found, the comprehensive version was not found to be inferior.^5^ Whilst Plunkett et al.,^5^ found some evidence that an information leaflet may help reduce maternal anxiety regarding fetal MRI, anxiety is complex, and the timing of information matters.

## A Substantial Number of Medical Imaging Patients, Including Those Undergoing Fetal MRI, Experience Anxiety

Data from the study by Plunkett et al.,^5^ indicated that around 50% of participants in each group felt anxious before the MRI, primarily about the MRI process, scan results and safety. Whilst their study did not use validated measure to assess anxiety, its findings align with other Australian research, indicating that around half of MRI patients experience anxiety,^11^ and emphasise the need for further research into appropriate education and support for patients to reduce anxiety.

## Measurement of Anxiety

Plunkett et al.^5^ acknowledge an important limitation of their study – anxiety was evaluated retrospectively only. Fakes et al.^6^ previously identified this and other issues in the measurement of anxiety – few studies examine anxiety in non‐cancer‐related imaging using validated measures and across timepoints. This is an important research gap as raised anxiety may be transitory: state anxiety refers to a transitory emotional state, or how a person feels at that particular point in time, whilst trait anxiety indicates how one generally feels or proneness to anxiety.^12^ Studies that have examined maternal psychological distress^13^, ^14^ have used various measures including the Perceived Stress Scale, Edinburgh Postnatal Depression Scale, Spielberger State–Trait Anxiety Inventory (STAI), the short‐form STAI: Y‐6 item and Visual Analogue Scales. A significant proportion of patients undergoing fetal MRI experience elevated psychological distress, ranging from 28% to 52% of participants.^13^ One of the few studies to examine anxiety trajectories across timepoints and a range of medical imaging procedures including MRI, found the prevalence of anxiety pre‐ and post‐procedure was similar, at around 50%, with the most common anxiety trajectory being persistent high anxiety (36%).^15^ Collectively, these findings suggest further interventions to reduce anxiety are needed.

## Factors Associated with Raised Anxiety

Factors found associated with raised anxiety prior to medical imaging include medical condition, imaging modality, gender, first time having the procedure and patient‐perceived health status.^11^ High pre‐procedure anxiety and low patient‐reported health status have been found to correlate with raised post‐procedure anxiety.^15^ In relation to fetal MRI, patients who reported they had used stress coping strategies and those who were accompanied by a supporting person at their appointment were found to have reduced anxiety and depressive symptoms, particularly in the setting of high‐risk pregnancies.^16^ Severity of the referral diagnosis has been found to affect pre‐MRI anxiety, especially in cases of fetal malformation,^17^ whilst a substantial number of women still experience psychological distress 1 year after a fetal MRI.^18^ Collectively, these findings, along with the paper by Plunkett et al.,^5^ emphasise the need for increased information and emotional support throughout the imaging process.

## A Pre‐Procedure Anxiety Screening Questionnaire Could Identify At‐Risk Patients

Research findings suggest that a pre‐procedure screening questionnaire may be useful to identify patients at risk of raised anxiety in relation to medical imaging,^15^ and more specifically fetal MRI.^14^ Universal screening for prenatal psychological distress and integrated cognitive‐behavioural interventions to optimise fetal neurodevelopment in cases of congenital heart disease^14^ has been suggested, with data indicating maternal psychological distress may impact fetal brain development, potentially leading to adverse neurodevelopment.^19^

## Effective Interventions to Reduce Procedural Anxiety Exist

Evidence‐based methods of reducing anxiety exist, including the provision of preparatory information and communication^20^, ^21^ and psychological approaches such as relaxation techniques (e.g. breathing exercises), cognitive coping strategies and emotional discussion.^22^ However, reviews^23^, ^24^ also highlight the need for further randomised controlled trials of preparatory information interventions to reduce anxiety.

## Early Education is Recommended

The authors identified a recurring theme from the qualitative component of their study – patients would prefer to receive the information leaflet from their doctor at the time of scan referral and not immediately before the scan.^5^ This led to their recommendation that providing education for referrers and ensuring they have information leaflets available to provide to patients at the time of referral could be a vital component in helping to reduce maternal anxiety.^5^ Further research into the timing of education and alternate methods of delivery would be beneficial to reducing maternal anxiety and improving patient experience.

## Providing Information to Patients About the Likely Timeframe and Process for Receiving Their Results is Warranted

It was found that most participants in all three groups agreed or strongly agreed they were anxious before the scan (50%) and were anxious about the results (over 60%).^5^ This finding is similar to that reported by Forshaw et al.^11^ who found that 48% of participants with raised anxiety felt most anxious or worried about the possible results. Whilst educational information provided pre‐procedure cannot directly address the nature of the individual imaging results, these studies highlight the importance of ensuring patients are well‐informed at the time of the initial referral regarding the timeframe and processes for receiving their results. This knowledge may further alleviate stress relating to the potential gap between patient expectations of quick results and the reality of the post‐procedure reporting process.

## The Way Forward: eHealth Holds Promise

Plunkett et al.^5^ investigated the influence of paper‐based information leaflets on patient education in a cohort of patients undergoing fetal MRI. Based on their findings they suggested that information leaflets should not be solely relied upon as a method to reduce anxiety. Further research examining the format and content of patient education is needed. In addition to printed information, eHealth interventions hold potential for delivering standardised information to patients and should be investigated for fetal MRI. eHealth can overcome geographical barriers, cater to individual needs and enable patients to review information at their own pace. eHealth is an area of increasing research, with research examining many aspects such as eHealth literacy,^25^ educational YouTube videos,^4^ and implementation strategies.^26^

Given the knowledge gaps in relation to the optimal timing and mode of information provision, further research on patients' preparation and experiences including anxiety is needed. Defining clear roles and responsibilities for both referring doctors and imaging service providers, in relation to information provision is increasingly important. eHealth systems offer feasible solutions, enabling patients to access information and communicate with healthcare providers.

## Conflict of Interest

The author declares that they have no competing interests.

## Data Availability

Data sharing is not applicable to this article as no datasets were generated or analysed during the current study.
